# The atypical chemokine receptor ACKR2 suppresses Th17 responses to protein autoantigens

**DOI:** 10.1038/icb.2014.90

**Published:** 2014-10-28

**Authors:** Chris AH Hansell, Lindsay M MacLellan, Rachel S Oldham, James Doonan, Katie J Chapple, Elinor JR Anderson, Christopher Linington, Iain B McInnes, Robert JB Nibbs, Carl S Goodyear

**Affiliations:** 1 Institute of Infection, Immunity and Inflammation, College of Medical Veterinary and Life Sciences, University of Glasgow, Glasgow, UK

## Abstract

Chemokine-directed leukocyte migration is a critical component of all innate and adaptive immune responses. The atypical chemokine receptor ACKR2 is expressed by lymphatic endothelial cells and scavenges pro-inflammatory CC chemokines to indirectly subdue leukocyte migration. This contributes to the resolution of acute inflammatory responses *in vivo*. ACKR2 is also universally expressed by innate-like B cells, suppressing their responsiveness to the non-ACKR2 ligand CXCL13, and controlling their distribution *in vivo*. The role of ACKR2 in autoimmunity remains relatively unexplored, although *Ackr2* deficiency reportedly lessens the clinical symptoms of experimental autoimmune encephalomyelitis induced by immunization with encephalogenic peptide (MOG_35–55_). This was attributed to poor T-cell priming stemming from the defective departure of dendritic cells from the site of immunization. However, we report here that *Ackr2*-deficient mice, on two separate genetic backgrounds, are not less susceptible to autoimmunity induced by immunization, and in some cases develop enhanced clinical symptoms. Moreover, ACKR2 deficiency does not suppress T-cell priming in response to encephalogenic peptide (MOG_35–55_), and responses to protein antigen (collagen or MOG_1–125_) are characterized by elevated interleukin-17 production. Interestingly, after immunization with protein, but not peptide, antigen, *Ackr2* deficiency was also associated with an increase in lymph node B cells expressing granulocyte-macrophage colony-stimulating factor (GM-CSF), a cytokine that enhances T helper type 17 (Th17) cell development and survival. Thus, *Ackr2* deficiency does not suppress autoreactive T-cell priming and autoimmune pathology, but can enhance T-cell polarization toward Th17 cells and increase the abundance of GM-CSF^+^ B cells in lymph nodes draining the site of immunization.

Chemokines play a major role in orchestrating innate and adaptive immune responses by controlling the migration of leukocytes using G protein-coupled chemokine receptors that decorate the surface of these cells.^1^ Alongside the large chemokine receptor family is a small subfamily of ‘atypical’ chemokine receptors, members of which bind chemokines with high affinity and specificity but appear incapable of classical chemokine receptor behavior.^2^ This subfamily is typified by ACKR2 (D6)^3^ a heptahelical membrane molecule structurally related to other chemokine receptors that binds a broad array of pro-inflammatory CC chemokines. In humans, ACKR2 is expressed by lymphatic endothelial cells, trophoblasts and some leukocyte populations.^4, 5, 6, 7, 8^ In mice, we have recently found that, among leukocytes, ACKR2 is highly restricted to innate-like B cells (IBCs) (that is, marginal zone and B1 B cells), and is the best unifying marker of these cells.^9^ IBCs serve key roles during homeostasis, autoimmunity and infection, and new properties of these cells continue to be defined. For example, recent work has revealed that B1 B cells generate ‘innate response activator’ B cells during inflammation that are dominant sources of the cytokine granulocyte-macrophage colony-stimulating factor (GM-CSF) in secondary lymphoid tissue.^10^

What sets ACKR2 and other atypical chemokine receptors apart is their inability to couple to signaling pathways activated after classical chemokine receptor engagement. Neither ACKR2-transfected cell lines nor primary ACKR2-expressing leukocytes migrate toward ACKR2 ligands.^2,9^ This, coupled with the ability of ACKR2 to continuously internalize chemokines,^11, 12, 13, 14, 15^ supports the concept that the principal function of ACKR2 is to act as a ‘professional’ chemokine scavenger that indirectly modulates leukocyte migration through chemokine removal. This model is used to explain phenotypes in challenged *Ackr2*-deficient mice that are often characterized by elevated chemokine abundance, exaggerated inflammation and increased immunopathology.^2,5,6,16, 17, 18, 19, 20, 21^ However, *Ackr2* deficiency also leads to cell-autonomous defects among IBCs (for example, increased responsiveness to the non-ACKR2 ligand CXCL13^9^) that are not dependent on loss of chemokine scavenging and could be linked to the ability of ACKR2 to regulate the subcellular distribution of β-arrestins, key regulators of G protein-coupled receptors like CXCR5.^14,15^ B1 cell distribution *in vivo* is profoundly dependent on engagement of CXCR5 by its ligand CXCL13,^22^ and *Ackr2-*deficient mice have fewer B1 B cells in their peritoneal cavity, omentum and spleen than wild-type (WT) animals.^9^ It is not clear whether this is because of the loss or redistribution of these cells, and the absence of suitable markers makes it difficult to distinguish B1 B cells from follicular B cells in other tissues, such as lymph nodes (LNs).

Chemokines are capable of influencing all the key steps that lead to the development of pathology in mouse models of autoimmunity, such as inflammation at the site of immunization, delivery of antigen to draining LNs, development of pathogenic lymphocytes and antibodies and the orchestration of immunopathology in the target tissue.^23,24^ Identifying how chemokine receptors contribute to the induction and maintenance of immunopathology has implications for the translation of chemokine receptor inhibition to the treatment of human disease.^25^ Only one study has examined the impact of *Ackr2* deficiency in a model of autoimmune disease,^26^ specifically experimental autoimmune encephalomyelitis (EAE) induced by immunization with a short peptide from rat myelin oligodendrocyte glycoprotein (MOG), referred to hereafter as MOG_35–55_. This study reported that, in contrast to the exaggerated inflammation seen in the absence of *Ackr2* in most other models, C57BL/6J *Ackr2*-deficient mice developed less brain inflammation and clinical symptoms of EAE than WT counterparts. This was attributed to the suppression of dendritic cell (DC) migration caused by excessive inflammation at the immunization site, and they reported a profound reduction in the proliferation of LN cells restimulated with MOG_35–55_ and reduced interferon-γ (IFNγ) release.^26^ Indeed, we have subsequently shown that, during inflammation, the absence of *Ackr2* is associated with the deposition of chemokines on skin lymphatic endothelial cells; perilymphatic accumulation of inflammatory leukocytes, including DCs; and concomitant ‘lymphatic congestion’.^27^

Here, using mice on two different genetic backgrounds, we report a detailed evaluation of the impact of *Ackr2* deficiency in four models of autoimmune disease: collagen-induced arthritis (CIA), collagen antibody-induced arthritis and EAE induced by immunization with MOG_35–55_ peptide or MOG_1–125_ protein. In none of these models did the absence of *Ackr2* decrease the severity of disease, and in some cases *Ackr2*-deficient mice developed worse clinical symptoms than WT animals. Moreover, T-cell priming was not diminished in *Ackr2*-deficient mice, and WT and *Ackr2*-deficient LN cells had comparable proliferation when restimulated with antigen. In fact, compared with WT, cells from the LN of *Ackr2*-deficient mice draining the site of immunization with protein (collagen or MOG_1–125_) but not peptide (MOG_35–55_) antigen showed an increased propensity to produce interleukin-17 (IL-17). Importantly, this was not an intrinsic T-cell phenomenon as chimeric studies demonstrated that WT and *Ackr2*-deficient T cells differentiated equally into T helper type 17 (Th17) cells when in the same animal. Interestingly, enhanced Th17 responses were associated with an increase in the abundance of B cells producing GM-CSF, a cytokine known to enhance Th17 cell development and survival.^28^ Thus, in the models we have tested, *Ackr2*-deficient mice are not less susceptible to autoimmunity and do not show suppressed T-cell responses, but can develop enhanced Th17 responses and greater numbers of GM-CSF^+^ B cells after immunization with protein autoantigen.

## RESULTS

### *Ackr2* is upregulated in arthritic mouse joints and suppresses the severity of CIA in DBA1/j mice

**Figure 1:**
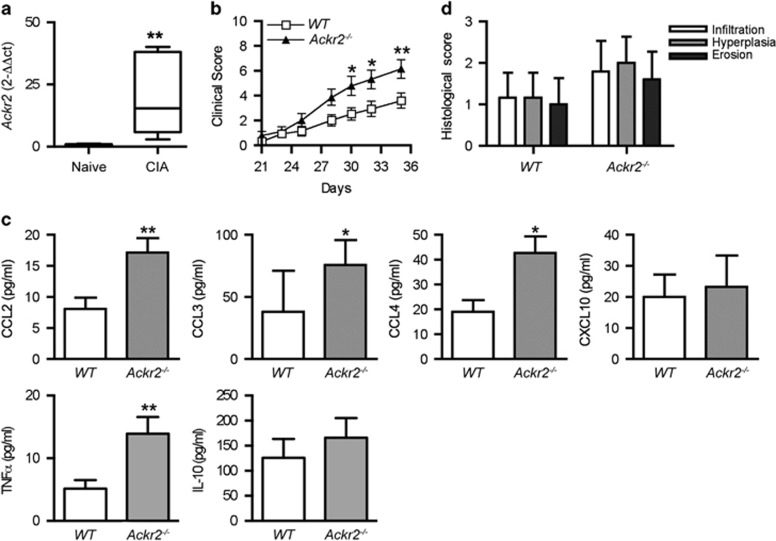
ACKR2 regulates chemokine abundance in arthritic joints and suppresses disease severity in CIA. (**a**) RNA was extracted from the knees of WT DBA/1j mice with established CIA (day 35) or age- and sex-matched naive littermate control mice (*n*=6). Quantitative reverse transcriptase-PCR (Q-RT-PCR) was performed to evaluate the level of *Ackr2* expression. *Ackr2* gene expression was normalized to GAPDH and the fold change in inflamed tissue calculated relative to naive tissue (set to 1). (**b**–**d**) CIA was induced in wild-type littermate control (WT) and *Ackr2*-deficient (*Ackr2*^−/−^) DBA/1j mice with 100 μg of bovine CII in complete Freund’s adjuvant. (**b**) Clinical score, with each point representing the pooled mean±s.e.m. scores of three experiments using *n*=29 mice of each genotype. Significance was determined by two-way repeated measures analysis of variance (ANOVA) with Bonferroni post tests. *P<0.05 and **P<0.01. (**c**) Knee patella and adjacent synovial tissue were harvested (*n*=6–8), cultured *ex vivo* for 4 h and the level of secreted chemokine and cytokines determined by ELISA. *P<0.05 and **P<0.01 (Mann–Whitney test). (**d**) Histological scores. Hematoxylin and eosin (H&E)-stained tissue sections were scored in a blinded manner for hyperplasia, infiltration and erosion. Bars show mean±s.d. of 5–6 per group.

By comparing healthy and arthritic knees from WT DBA1/j mice, we found that *Ackr2* transcripts were significantly upregulated in the target tissue of inflammatory arthritis (Figure 1a). We considered whether loss of the anti-inflammatory activity of ACKR2 at this site might have a more pronounced effect on the development of autoimmune disease than it is reported to have in the brain.^26^ To explore this, we backcrossed *Ackr2*-deficient C57BL/6J mice onto DBA/1j and monitored development of arthritis in a large cohort of animals after immunization with bovine type II collagen, using WT DBA1/j littermate mice as controls. In contrast to previous observations in the EAE model,^26^ the absence of *Ackr2* resulted in a statistically significant increase in the clinical symptoms of arthritis (Figure 1b), and a substantial increase (*P*<0.05) in the cumulative clinical score of *Ackr2*-deficient mice (24.2±3.6, mean±s.d.) compared with WT mice (13.7±2.6). There was, however, no change in the time at which these animals first developed symptoms (incidence of disease) (data not shown). *Ackr2*-deficient mice often have elevated levels of ACKR2-binding chemokines in inflamed tissues.^16,17,20^ Consistent with this, two- to threefold more ACKR2-binding chemokines CCL2, CCL3 and CCL4, but not non-ACKR2-binding chemokine CXCL10, were released from explants of patella and adjacent synovial tissue from arthritic *Ackr2*-deficient mice, compared with equivalent preparations from arthritic WT animals (Figure 1c). Furthermore, a corresponding increase in the release of tumor necrosis factor-α was observed in the *Ackr2*-deficient patella and adjacent synovial tissue explants, whereas the levels of IL-10 were unchanged (Figure 1c). Histological analysis revealed a trend toward an increase in pathological changes in the inflamed joints in *Ackr2*-deficient mice, but this failed to achieve statistical significance (Figure 1d). Thus, ACKR2 is locally upregulated in response to the development of inflammation in the mouse joint, controls the levels of inflammatory CC chemokines in the diseased tissue and suppresses the clinical symptoms of arthritis.

### Loss of ACKR2 does not alter anti-collagen antibody levels in CIA or the development of antibody-driven inflammatory arthritis

**Figure 2:**
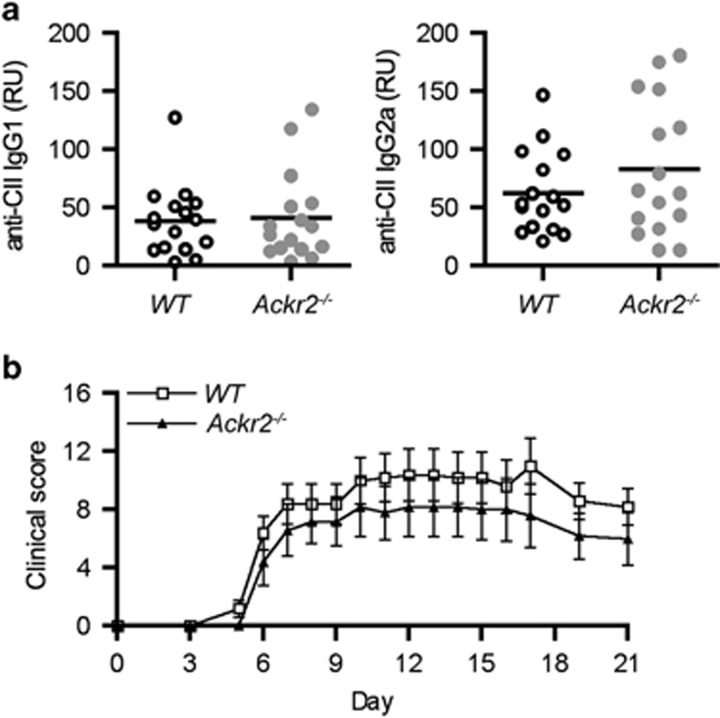
*Ackr2* deficiency has no impact on the generation of anti-collagen antibodies in CIA and or on antibody-induced arthritis. (**a**) Serum levels of anti-collagen mouse IgG1 and IgG2a in arthritic wild-type littermate control (WT) and *Ackr2*-deficient (*Ackr2*^−/−^) DBA/1j mice (*n*=16) at 35 days after the first collagen immunization. (**b**) Collagen antibody-induced arthritis (CAIA) was induced in wild-type (WT) and *Ackr2*-deficient (*Ackr2*^−/−^) DBA/1j mice with each animal assessed every 1 to 2 days for clinical symptoms of arthritis. Each point represents the mean±s.e.m. scores of *n*=5 mice. Significance was determined by two-way repeated measures analysis of variance (ANOVA).

Loss of ACKR2 did not affect the level of subclass-specific anti-type II collagen (CII) antibodies produced during CIA (Figure 2a). However, we thought that the increased clinical disease in *Ackr2*-deficient mice in CIA might be the result of an altered innate response in the joint to these pathogenic antibodies. To investigate this directly, we examined the impact of *Ackr2* deficiency on the development of anti-collagen antibody-induced arthritis. Pathology in this model relies heavily on innate effector mechanisms involving neutrophils, macrophages, Fc receptors, complement and inflammatory chemokines and cytokines.^29^ However, loss of ACKR2 had no impact on the development or maintenance of arthritic disease in this model (Figure 2b). Thus, the enhanced disease observed in *Ackr2*-deficient mice in the CIA model was unlikely to be the result of exaggerated responses to anti-collagen antibodies.

### Increased IL-17 production in LNs draining arthritic joints in *Ackr2*-deficient mice

**Figure 3:**
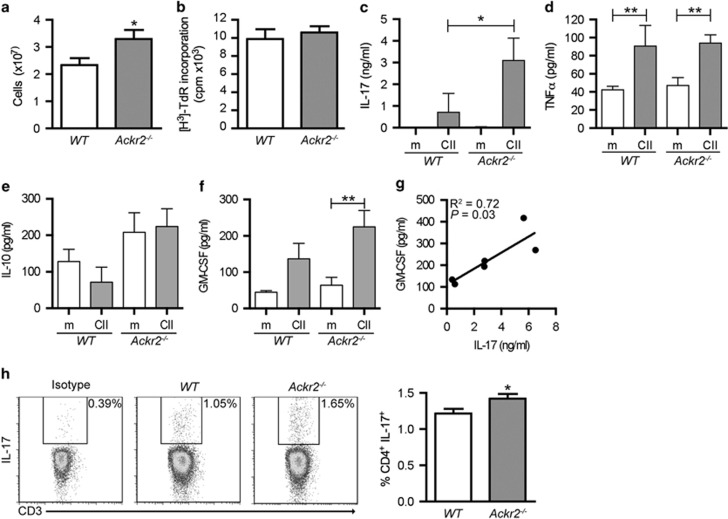
Arthritic *Ackr2*-deficient mice have enlarged draining LNs capable of greater collagen-induced IL-17 production. Draining LNs (*n*=6–10) were collected from wild-type (WT) and *Ackr2*-deficient (*Ackr2*^−/−^) DBA/1j mice 35 days after collagen immunization. (**a**) Number of LN cells retrieved. (**b**–**g**) Draining LN cells (3 × 10^5^ cells per well) were stimulated *ex vivo* with or without antigen (CII, 60 μg ml^−1^). (**b**) Ag-induced proliferation (that is, CII minus medium alone), as determined by incorporation of tritiated thymidine ([H^3^]-TdR). (**c**–**f**) Concentration of IL-17, tumor necrosis factor-α (TNFα), IL-10 and GM-CSF in the medium after stimulation with antigen (CII) or medium alone (m). Significance was determined by one-way analysis of variance (ANOVA) with Bonferroni multiple comparison post tests. *P<0.05 and **P<0.01. (**g**) Correlation between the concentration of GM-CSF and IL-17. Significance was determined by Spearman’s rho test. (**h**) Representative results from IL-17 intracellular flow cytometry and a bar graph showing the percentage of LN cells that were CD4^+^IL17^+^ cells. A repeat experiment yielded similar results. Significance was determined by *t*-test. *P<0.05.

Next, we examined whether there was any evidence of altered T-cell responses in arthritic *Ackr2*-deficient mice by analyzing inguinal LNs draining the arthritic hind legs of WT and *Ackr2*-deficient mice 35 days after the induction of disease. *Ackr2* deficiency had no impact on the cellularity of these LNs in resting mice (data not shown), but more cells were retrieved from the inflamed inguinal LNs of arthritic *Ackr2*-deficient mice than from WT counterparts (Figure 3a). There was no difference in the proliferative response of these cells to CII stimulation (Figure 3b), but, compared with WT, *Ackr2*-deficient cells released approximately fivefold more IL-17 into the medium after CII stimulation (Figure 3c). In contrast, there was no difference in CII-induced tumor necrosis factor-α or IL-10 production by LN cells harvested from WT and *Ackr2*-deficient mice (Figures 3d and e). However, *Ackr2*-deficient cells produced significantly more GM-CSF after stimulation with CII (Figure 3f) and, interestingly, in the CII-stimulated *Ackr2*-deficient cultures, the level of IL-17 significantly correlated with GM-CSF abundance (*r*^2^=0.72, *P*=0.03; Figure 3g). Moreover, compared with WT, the inguinal LNs draining the inflamed joints of *Ackr2*-deficient mice showed a small, but statistically significant, increase in the proportion of CD4^+^ T cells capable of producing IL-17 after stimulation *in vitro* with phorbol 12-myristate 13-acetate/ionomycin, indicative of an increased generation of Th17 cells in these mice (Figure 3h). This bias was restricted to CD4^+^ IL-17-producing cells as no significant differences were observed in the proportion of IL-17^+^ CD8^+^ or γδ T cells (data not shown and Supplementary Figure 1). Thus, *Ackr2*-deficient mice developed an effective T-cell response to CII, and, by day 35, their CD4^+^ T-cell population had a significantly greater Th17 component.

### ACKR2 suppresses the development of Th17 cells during the initiation of the arthritogenic immune response

**Figure 4:**
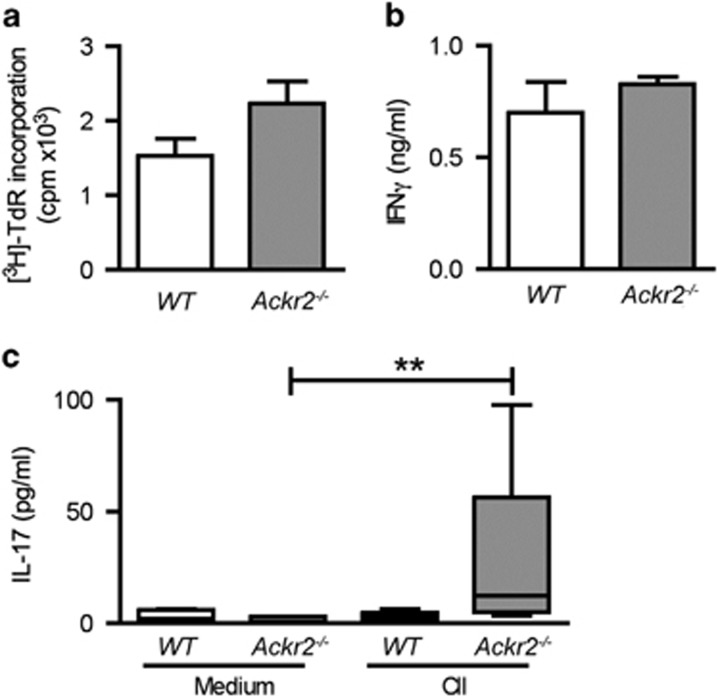
*Ackr2* deficiency leads to an increased Th17 response during the induction of arthritis. At 8 days after challenge with CII, draining LNs were harvested from WT littermate control (WT) and *Ackr2*-deficient (*Ackr2*^−/−^) DBA/1j mice (*n*=5) and the recall immune response evaluated. Single-cell suspensions of LN cells (3 × 10^5^ cells per well) were stimulated with CII (60 μg ml^−1^) or medium alone and assessed for (**a**) specific proliferation (that is, proliferation after CII stimulation minus proliferation in medium alone) or (**b**, **c**) the levels of IFNγ and IL-17 in the medium. **P<0.01 by Kruskal–Wallis test with Dunn’s multiple comparison post test. A repeat experiment yielded similar results.

The resistance of *Ackr2*-deficient mice to EAE was ascribed to a defect in T-cell priming,^26^ but our data showed that there was a plentiful supply of collagen-specific lymphocytes in arthritic *Ackr2*-deficient mice. This suggested that loss of ACKR2 might not suppress the priming of antigen-specific T cells in the CIA model. To specifically evaluate this, WT and *Ackr2*-deficient DBA1/j mice were immunized with CII in complete Freund’s adjuvant and draining LNs were harvested on day 8, the time point selected in previous work after immunization with MOG_35–55_.^26^ However, in marked contrast to the poor T-cell responses reportedly elicited by MOG_35–55_,^26^ proliferation and IFNγ secretion of antigen-stimulated lymphocytes were not suppressed in *Ackr2*-deficient LNs (Figures 4a and b). In fact, and in line with the data from the arthritic mice (Figure 3), a significant increase in the production of IL-17 was observed after stimulation of *Ackr2*-deficient lymphocytes with CII and, strikingly, this cytokine was not detectably induced when WT lymphocytes were used (Figure 4c). Thus, loss of ACKR2 does not impair the ability to detect and respond to antigenic challenge in this model, and it actually enhances the initial Th17 response.

### MOG_35–55_-induced EAE is not suppressed by the absence of ACKR2

**Figure 5:**
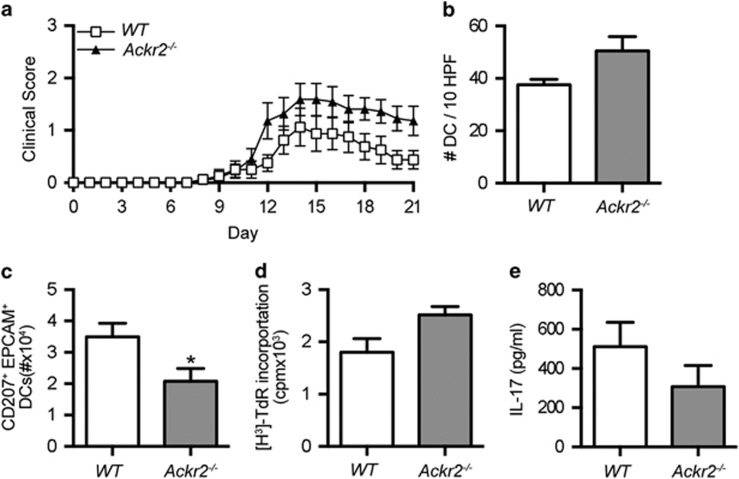
*Ackr2* deficiency does not impair disease severity in the MOG_35–55_ peptide-induced model of EAE or result in increased IL-17 production. EAE was induced in WT littermate control (WT) and *Ackr2*-deficient C57BL/6 mice (*Ackr2*^−/−^) by immunization with MOG_35–55_ (100 μg) in complete Freund’s adjuvant (4 mg ml^−1^ *Mycobacterium tuberculosis* H37RA) and treatment on days 0 and 2 with 200 ng of pertussis toxin (PTX). (**a**) All animals were assessed daily for the development of clinical symptoms of disease. Each point represents the mean±s.e.m. scores for *n*=5 mice. A repeat experiment yielded similar results. (**b**) Injection site skin was harvested on day 3 and the number of CD207^+^ DCs in 10 high-power fields (HPFs) per mouse determined. (**c**–**e**) Draining LNs were harvested on day 3 or day 11 and single-cell suspensions were evaluated for (**c**) the absolute number of CD207^+^EPCAM^+^ DCs (day 3) or (**d**, **e**) 3 × 10^5^ cells per well stimulated with MOG_35–55_ (30 μg ml^−1^) (day 11) and assessed for (**d**) specific proliferation (proliferation after MOG_35–55_ stimulation minus proliferation in medium alone) or (**e**) the level of IL-17 in the medium. Significance was determined by *t-*test. *P<0.05.

The absence of suppressed T cell priming after immunization with collagen led us to re-evaluate the role ACKR2 during EAE induction in C57BL/6J mice. In stark contrast to the previous work,^26^ we found that *Ackr2* deficiency offered no protection against disease after immunization with MOG_35–55_ peptide (Figure 5a). We were also unable to observe a difference in the accumulation of DCs at the site of immunization in *Ackr2*-deficient mice 3 days after injection (Figure 5b), although in the draining LN at this time there was a significant decrease in the number of CD207^+^ EPCAM^+^ migratory DCs in *Ackr2*-deficient mice (Figure 5c) that was also evident at day 21 (data not shown). Nonetheless, no differences were detected in lymphocyte proliferation on day 11 or day 21 when the *Ackr2*-deficient and WT inguinal LN cells were stimulated *ex vivo* with MOG_35–55_ (Figure 5d, and data not shown). Importantly, in this setting *Ackr2*-deficient cells did not produce more IL-17 than their WT counterparts (Figure 5e). Thus, although loss of ACKR2 did reduce the number of migratory DCs in the LN draining the site of MOG_35–55_ immunization, it did not hamper the ability of T cells to respond to antigenic challenge and, in contrast to immunization with CII protein, there is no evidence of an enhanced Th17 response.

### MOG_1–125_-induced EAE is not suppressed by the absence of ACKR2 but Th17 responses are enhanced

**Figure 6:**
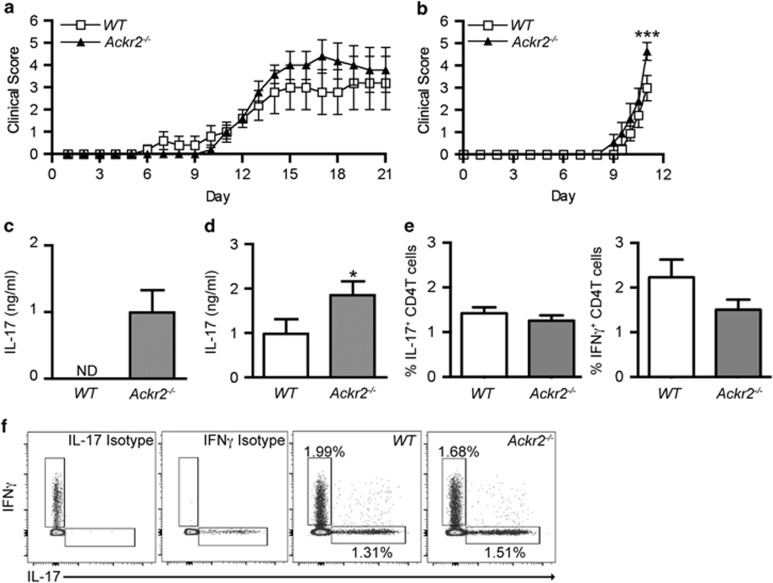
*Ackr2* deficiency leads to increased IL-17 production after the induction of EAE in C57BL/6 and DBA/1j mice, and has minimal effect on disease severity. (**a**–**d**) EAE was induced in WT littermate control (WT) and *Ackr2*-deficient (*Ackr2*^−/−^) C57BL/6 (**a**, **c**) or DBA/1j (**b**, **d**) mice by immunization with MOG_1–125_ protein (50 μg in 3 mg ml^−1^ *Mycobacterium tuberculosis* H37RA CFA). (**a**, **b**) Animals evaluated daily for clinical symptoms of disease. In (**a**), each point represents the means±s.e.m. (*n*=5 mice per genotype). A repeat experiment generated a similar result. In (**b**), the pooled mean±s.e.m. scores of two experiments are shown (*n*=10–11 mice per genotype). Significance was determined by two-way repeated measures analysis of variance (ANOVA) with Bonferroni post tests. ***P<0.001. (**c**, **d**) At 21 (*n*=5) (**c**) or 11 days (*n*=10–11) (**d**) after immunization, 3 × 10^5^ cells per well from LNs draining the site of immunization were stimulated *ex vivo* with MOG_1–125_ (60 μg ml^−1^) or medium alone and the concentration of IL-17 in the medium was determined. *P<0.05 by Mann–Whitney test. ND=not detected. (**e**, **f**) Mixed bone marrow chimeric mice (WT:*Ackr2*^−/−^) were immunized with MOG_1–125_ protein (50 μg in 3 mg ml^−1^ *Mycobacterium tuberculosis* H37RA CFA), draining inguinal LNs were harvested on day 15 and the number of CD4^+^TCRβ^+^CD45.1^+^ (WT) or CD4^+^TCRβ^+^CD45.1^−^ (*Ackr2*^−/−^) IL-17A^+^ and IFNγ^+^ T cells determined. (**e**) Bar graphs showing the percentage of CD4^+^TCRβ^+^ cells that were IL17^+^ or IFNγ^+^. (**f**) Representative flow cytometry plots pregated on live singlet CD4^+^TCRβ^+^CD45.1^+^ (WT) or CD4^+^TCRβ^+^CD45.1^−^ (*Ackr2*^−/−^).

*Ackr2* deficiency did not affect the Th17 response in the peptide-driven C57BL/6 model of EAE, but enhanced this response in the DBA1/j collagen-driven model of CIA. This led us to question the role of ACKR2 and/or genetic background in protein-driven priming of T-cell responses. As above, when EAE was induced in WT and *Ackr2*-deficient mice on the C57BL/6 or DBA1/j strain using MOG_1–125_ protein, *Ackr2* deficiency did not offer any protection against disease (Figures 6a and b). In fact, there was a small, but statistically significant, increase in the clinical symptoms of disease in *Ackr2*-deficient DBA1/j mice 11 days after immunization (Figure 6b), although histological examination of the brains of these animals failed to reveal any change in leukocyte infiltrate (data not shown). The severe EAE induced in *Ackr2*-deficient DBA1/j mice by MOG_1–125_ meant that, under the terms of our license, animals had to be killed on day 11. As in the other models, no differences were detected in lymphocyte proliferation when *Ackr2*-deficient and WT inguinal LN cells were stimulated *ex vivo* with MOG_1–125_, and IFNγ production was also unaffected (data not shown), but notably *Ackr2*-deficient cells from both C57BL/6 and DBA1/j mice produced significantly more IL-17 than their WT counterparts (Figures 6c and d). In further experiments, MOG_1–125_-immunized WT and *Ackr2*-deficient C57BL/6 mice were treated with pertussis toxin on days 0 and 2 (Supplementary Figure 2). As before, these animals developed comparable clinical symptoms and elevated Th17 responses. Thus, loss of ACKR2 does not suppress the pathogenicity of protein autoantigens (collagen and MOG_1–125_) and is consistently associated with enhanced Th17 responses, regardless of genetic background.

### Enhanced Th17 responses in *Ackr2*-deficient mice are not due to intrinsic T-cell defects

Although there is no evidence that T cells express ACKR2 protein in mice,^9^ transcripts encoding ACKR2 can be detected in mouse T cells activated in culture, and in samples of mouse secondary lymphoid tissues that have been enriched for T cells.^8^ It was possible therefore that *Ackr2* expression by T cells played a role in regulating the generation of Th17 cells. To examine this, we generated chimeric mice carrying both WT and *Ackr2*-deficient hematopoietic cells to allow us to evaluate whether WT and *Ackr2*-deficient T cells activated in the same host differed in their ability to generate Th17 cells. These animals were immunized with MOG_1–125_ protein, and Th1 and Th17 cells identified in the WT and *Ackr2*-deficient CD4^+^ T-cell populations in the inguinal LN by using intracellular cytokine staining to identify T cells capable of producing IFNγ or IL-17, respectively. Under these circumstances, there was no difference in the percentage of WT and *Ackr2*-deficient T cells that could be classified as Th1 or Th17 cells (Figures 6e and f). Thus, ACKR2 in T cells does not detectably influence their ability to develop into Th17 cells.

### Enhanced Th17 responses in *Ackr2*-deficient mice are associated with increased numbers of GM-CSF^+^ B cells in draining LN

**Figure 7:**
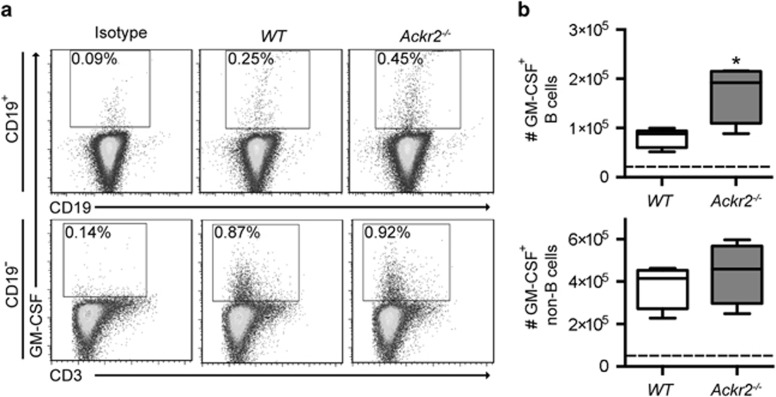
*Ackr2* deficiency leads to increased numbers of GM-CSF^+^ B cells in draining LNs after immunization with MOG_1–125_ protein. EAE was induced in WT littermate control (WT) and *Ackr2*-deficient (*Ackr2*^−/−^) C57BL/6 mice (*n*=4) by immunization with MOG_1–125_ protein (50 μg in 3 mg ml^−1^ *Mycobacterium tuberculosis* H37RA CFA), draining LNs harvested on day 8, and the number of GM-CSF^+^ CD19^+^ B cells determined. (**a**) Representative flow cytometry plots that were pregated on live IgD^+^IgM^+^ singlets (top panels) and live IgD^−^IgM^−^CD19^−^ singlets (bottom panels), and the percentage of GM-CSF^+^ CD19^+^ B cells and GM-CSF^+^ CD19^−^ non-B cells respectively are shown. (**b**) Box and whisker plots, where the boxes represent the 25th to 75th percentiles, the lines within the boxes represent the median and the lines outside the boxes represent the 5th and 95th percentiles, show the absolute number of GM-CSF^+^ B cells (top plot) and GM-CSF^+^ non-B cells (bottom plot) in the draining LNs. The dotted line represents the isotype control level. A repeat experiment yielded similar results. *P<0.05 by Mann–Whitney test.

ACKR2 is expressed by IBCs, including rare B1 cells in LN, and the B1 B-cell compartment is perturbed in resting *Ackr2*-deficient mice.^9^ B1a B cells have been shown to give rise to a population of cells in the spleen during inflammation that are a dominant source of GM-CSF.^10^ GM-CSF, via IL-6 and IL-23, enhances Th17 cell development and survival.^28^ Thus, we considered whether during the initiation of an immune response, GM-CSF^+^ B cells were present in draining LN, and whether their abundance was affected by *Ackr2* deficiency. LNs were harvested 8 days after immunization of WT and *Ackr2*-deficient C57BL/6 mice with MOG_35–55_ or MOG _1–125_, and GM-CSF expression assessed by intracellular cytokine staining (Figure 7, and data not shown). Small numbers of GM-CSF^+^ B cells were detected in the LNs of WT mice, and the number retrieved was similar irrespective of whether they had been immunized with peptide (66 107±27 981 GM-CSF^+^ B cells; mean±s.d.) or protein (79 406±18 994 GM-CSF^+^ B cells; mean±s.d.). Interestingly, however, draining LNs from *Ackr2*-deficient mice immunized with MOG_1–125_ protein contained significantly more GM-CSF^+^ B cells than their WT counterparts, whereas no increase was observed when MOG_35–55_ peptide was used. No differences were detected in the levels of GM-CSF^+^ non-B-cell populations in *Ackr2*-deficient and WT counterparts (Figure 7). Surface immunophenotyping revealed that these cells, whether WT or *Ackr2* deficient, were mostly CD138^−^ CD43^−^ IgM^hi^ IgD^hi^, although approximately a quarter had much lower levels of surface IgM (data not shown). Thus, enhanced Th17 responses of *Ackr2*-deficient mice are associated with an increase in the number of B cells making GM-CSF, a cytokine that controls the development and survival of Th17 cells.

## DISCUSSION

In the models we have examined, and using mice on two different genetic backgrounds, we find that deletion of *Ackr2* does not suppress T-cell priming or provide protection against autoimmunity. It can however enhance Th17 responses and the abundance of GM-CSF^+^ B cells in LNs after immunization with protein, but not peptide, autoantigen.

The responses that we observed in WT and *Ackr2*-deficient C57BL/6j mice after immunization with MOG_35–55_ clearly differ from those reported by Liu *et al.*,^26^ even though very similar immunization strategies were used. In their study, the reduced clinical and histopathological signs of disease seen in *Ackr2*-deficient mice from day 14 after immunization with MOG_35–55_ were attributed to dysregulated inflammation at the site of immunization causing defective DC migration from the skin.^26^ We have found that *Ackr2* deficiency can lead to ‘lymphatic congestion’ and the impaired movement of antigen-presenting cells from the skin,^27^ and shown here that DC migration to draining LNs is attenuated in *Ackr2*-deficient mice after immunization with MOG_35–55_. Thus, ACKR2 does allow optimal DC trafficking to LN from inflamed skin. Where our studies differ is in the impact of *Ackr2* deficiency on T-cell priming and disease course. Liu *et al.*^26^ reported that LN cells draining the site of immunization in *Ackr2*-deficient mice were virtually unable to proliferate after restimulation with antigen *ex vivo*, in stark contrast to the strong responses seen when LN cells from immunized WT mice were used. We saw robust proliferative responses in *Ackr2*-deficient mice after immunization with MOG_35–55_ (or, indeed, collagen or MOG_1–125_) and these responses were no weaker than those seen in WT controls. Although EAE was successfully induced in both studies, we failed to see the divergence in the clinical scores of WT and *Ackr2*-deficient C57BL/6j mice 14 days after immunization with MOG_35–55_ that was reported in the previous work. This was also the case after immunization of WT and *Ackr2*-deficient C57BL/6j mice with MOG_1–125_. There was in fact a small increase in EAE score when *Ackr2*-deficient mice on a DBA/1j background were compared with WT DBA/1j mice, and *Ackr2*-deficient DBA/1j mice also developed worse arthritis than WT animals during CIA. We have yet to resolve the differences between our study and those of Liu *et al.*,^26^ but our findings clearly challenge the notion that ACKR2 facilitates the development of pathogenic T cells in mouse models of autoimmune disease.

*Ackr2* expression was upregulated in the knees or central nervous system of DBA1/j mice with CIA or EAE, respectively (Figure 1, and data not shown), perhaps in an effort to counteract ongoing inflammation. The source of this *Ackr2* is not clear and there are no effective anti-ACKR2 antibodies available with which to identify and localize ACKR2-expressing cells by immunohistochemistry in mouse tissues. However, as ACKR2 is found in subsets of leukocytes, lymphatic endothelial cells and epithelial cells in resting and inflamed human tissues, including those affected by autoimmune disease,^4,8,30,31^ it seems likely that these cell types are responsible for *Ackr2* expression in inflamed tissues in mice. However, it remains unclear whether the enhanced *Ackr2* expression seen during autoimmune inflammation is either because of its presence on infiltrating leukocytes or its induction on resident cells by signals that are still to be defined. Synovial explants from arthritic *Ackr2*-deficient DBA1/j mice released more ACKR2-binding chemokine (CCL2, 3 and 4) than WT counterparts, whereas levels of the non-ACKR2 ligand CXCL10 were unaffected. These data suggest that loss of ACKR2-mediated chemokine scavenging in the target tissue might contribute to the enhanced clinical symptoms seen in *Ackr2*-deficient DBA1/j mice in CIA. However, the enhanced Th17 responses alone could be responsible, and this is supported by the fact that clinical symptoms of disease are unaffected by *Ackr2* deficiency in models that exclusively examine the effector arm of disease development (such as, anti-collagen antibody-induced arthritis in DBA1/j mice; EAE in C57BL/6J mice driven by transfer of MOG_35–55_-primed T cells^26^). Thus, it is unlikely that ACKR2 in the joint or central nervous system plays any significant role in regulating the inflammation that develops in these tissues in the models that we have used. The amount of ACKR2 expressed at these sites may be insufficient to scavenge enough chemokine to exert any inhibitory effect on disease progression in the presence of autoreactive T cells and autoantibodies, and joints and brain are relatively poor sources of ACKR2 compared with skin, lung, gut and placenta where the effects of *Ackr2* deficiency are most apparent.^2,5,6,16, 17, 18, 19, 20, 21^

One consistent feature of our study was the enhanced Th17 responses seen in *Ackr2*-deficient mice after immunization with protein (collagen or MOG_1–125_) but not peptide (MOG_35–55_). The data from the analysis of mixed bone marrow chimeras, in which WT and *Ackr2*-deficient T cells developed equally well into Th17 cells in response to protein antigen, suggest that this was not due to an intrinsic T-cell defect arising from loss of *Ackr2*. It may instead be due to differences in antigen presentation, and interestingly, peptide and protein can be presented by distinct antigen-presenting cells.^32,33^ In general terms, peptides are most efficiently presented to naive T cells by DCs, but proteins can be processed and presented by other cells, most notably B cells. Indeed, a dependence on B-cell presentation for T-cell priming has been demonstrated in protein-induced models of both arthritis and EAE.^34,35^ Moreover, B-cell depletion leads to diametrically opposed outcomes in the two modes of immunization: exacerbating peptide-driven EAE but suppressing protein-induced disease.^35^ In the protein model, high titers of pathogenic MOG-specific antibody are generated and B cells are capable of efficiently priming encephalogenic Th1 and Th17 cells. In the peptide model, MOG-specific antibody is less important, DCs are thought to dominate T-cell priming and IL-10-producing regulatory B cells can suppress disease.^36^ Thus, in *Ackr2*-deficient mice immunized with MOG_1–125_ or collagen, it appears likely that B cell-mediated T-cell priming overcomes any ‘lymphatic congestion’ or reduction in DC-mediated T-cell priming that might conceivably be occurring in these animals.

An increased dependence on B cells for T-cell priming in *Ackr2*-deficient mice might be responsible for the increase in IL-17-producing cells that we see in LNs after their immunization with collagen or MOG_1–125_ protein. In this regard, it is intriguing that IBC (that is, marginal zone and B1 B cells) are the principal leukocytes that express ACKR2 in mice.^9^ These cells exhibit a number of properties that might contribute to the generation of Th17 responses. For example, compared with follicular B cells, antigen-loaded IBCs are particularly adept at priming naive T cells,^37^ and B1 B cells are known to bias T-cell differentiation toward a Th17 phenotype *ex vivo.*^38^ Moreover, B1 B cells can give rise to GM-CSF^+^ B cells in the spleen,^10^ and during inflammation these cells appear to be a dominant leukocytic source of this cytokine that is known to enhance Th17 cell development and survival.^28^ Our work now demonstrates that GM-CSF^+^ B cells are present in LNs draining immunized skin, and their abundance is regulated by ACKR2 after immunization with protein autoantigens. The precise identity and origin of these cells remains uncertain, and their surface immunophenotype is clearly distinct from the GM-CSF^+^ B cells that can be generated in the spleen to provide protection against microbial sepsis that express CD43 and CD138, among other markers.^10^ Nonetheless, we speculate that GM-CSF^+^ B cells contribute, at least in part, to the enhanced Th17 responses seen in *Ackr2*-deficient animals and experiments are underway to explore this possibility. Mechanistically, their increased abundance in inflamed *Ackr2*-deficient LN may be linked to cell-autonomous changes in the migratory properties of these cells, or their precursors. *Ackr2* deficiency enhances the migration of B1 B cells toward the non-ACKR2 ligand CXCL13,^9^ the principal chemokine involved in the localization of B cells, including IBCs.^22^ Through its receptor CXCR5, it recruits B cells into follicles in secondary lymphoid tissues and regulates their intrafollicular motility. It is conceivable that enhanced CXCL13 responsiveness alters the recruitment or positioning of certain subsets of B cells in *Ackr2*-deficient mice before or during inflammation, and that this favors the development of GM-CSF^+^ B cells. These possibilities are currently being addressed in our laboratory.

In conclusion, ACKR2 can regulate T-cell priming and GM-CSF-producing B cells during the induction of arthritic and neuropathic autoimmunity in mice, and its deletion leads to subtle changes in the development of disease. The precise nature of this regulation depends on whether peptide or protein antigen is used to induce the disease, clearly demonstrating the importance of using multiple models when assessing the role of specific genes in autoimmunity. Given the regulatory role of ACKR2 during inflammation and T-cell priming, it is important that studies are now undertaken to further investigate the expression and regulation of ACKR2 in the inflamed tissues and draining LNs of patients with autoimmune disease, and it remains to be seen whether ACKR2-mediated chemokine scavenging can influence disease progression in humans. A greater understanding of these aspects of ACKR2 biology could potentially lead to the development of new treatments in which the artificial elevation of ACKR2 in chronically inflamed tissues could be used to broadly suppress chemokine-driven inflammation.

## METHODS

### Mice

*Ackr2*-deficient mice (DBA1/j (F10) and C57BL/6J (F11)) and WT counterparts were housed under specific pathogen-free conditions at Glasgow University’s Central Research Facility. Animal studies were approved by the University of Glasgow Ethical Review Process and licensed by the UK Home Office.

### Induction and assessment of arthritis

CIA was induced in DBA/1j mice with 100 μg of bovine CII emulsified in complete Freund’s adjuvant (MD Biosciences, Zürich, Switzerland) at the base of the tail on day 0, and boosted on day 21 with an intraperitoneal injection of CII in phosphate-buffered saline (PBS), as previously described.^39^ Collagen antibody-induced arthritis was induced by intravenous injection of 2 mg of collagen antibody cocktail (Chondrex, Redmond, WA, USA). After 3 days, lipopolysaccharide (50 μg per mouse, Chondrex) was injected intraperitoneally. Mice were scored by a ‘genotype-blinded’ observer for clinical signs of disease, as previously described.^39^ Hind paws were histologically scored for inflammation and joint damage, as previously described.^40^ In brief, 0=healthy, 1=mild, 2=moderate and 3=severe. A genotype-blinded observer scored three sections per knee and the mean score per group was calculated.

### Quantitative PCR

RNA was extracted using RNeasy columns with DNAse treatment (Qiagen, Manchester, UK), complementary DNA generated (AffinityScript (Agilent, Santa Clara, CA, USA)) and quantitative PCR done using *Ackr2*-specific Taqman assay (mm0044555_m1) (Life Technologies, Paisley, UK) on a Prism 7900HT (Life Technologies). Glyceraldehyde-3-phosphate dehydrogenase (GAPDH)-specific probes were used to normalize *Ackr2* expression. Analysis used the relative quantitation ΔΔ^−2^ CT method to give the relative quantification (fold change) value, with naive tissue as calibrators set to 1.

### Synovial tissue explants

Patella and adjacent synovium were dissected and immediately incubated in complete RPMI for 4 h at 37 °C. The supernatant was harvested and assayed for chemokines, as described below.

### Restimulation of LN cells

Inguinal LN cells were harvested and single-cell suspensions prepared by enzymatic digestion using 1 mg ml^−1^ collagenase D (Roche, Burgess Hill, UK) in Hanks’ balanced salt solution without calcium and magnesium. Cells were cultured in triplicate in 96-well round-bottomed plates at 3 × 10^5^ cells per well in complete Dulbecco’s modified Eagle’s medium. Cells were restimulated with medium, 60 μg ml^−1^ of bovine tracheal cartilage (Sigma-Aldrich, Gillingham, UK) (in CIA model) or 30 μg ml^−1^ of the MOG_35–55_ peptide or MOG_1–125_ protein (in EAE model). Proliferation was analyzed at 88 h by [^3^H]Thymidine (GE Healthcare, Little Chalfont, UK) incorporation during the last 16 h of culture.

### Luminex and enzyme-linked immunosorbent assay (ELISA)

Cytokines, chemokines and anti-collagen antibody (Ab) levels were quantified by Luminex (Life Technologies) or ELISA using appropriately diluted sera or culture supernatants. Reagents for the quantification of mouse chemokines, IFNγ, IL-17 and tumor necrosis factor-α were from Biosource (Life Technologies), and assays were performed according to the manufacturer’s instructions. Anti-collagen Ab titers of individual sera were evaluated using ELISA-grade collagen (Chondrex) and detected with horseradish peroxidase-conjugated anti-mouse IgG1 or IgG2a (Southern Biotech, Birmingham, AL, USA). Total IgG was determined using an unlabeled anti-mouse IgG capture Ab and detected with horseradish peroxidase-conjugated anti-mouse IgG1 or IgG2a. Antibody ELISAs were developed using *o*-phenylenediamine dihydrochloride substrate (Sigma-Aldrich).

### Flow cytometry

LN cells were harvested and single-cell suspensions prepared by enzymatic digestion using 1 mg ml^−1^ collagenase D (Roche) in Hanks’ balanced salt solution without calcium and magnesium. These cells were resuspended in FACS buffer (PBS, 1% fetal calf serum, 0.02% sodium azide and 5 mM EDTA). 1–3 × 10^6^ cells per well of a 96-well round-bottomed plate were incubated with 50 μl of a 5 μg ml^−1^ solution of FC block (BD Biosciences, Oxford, UK) for 15 min on ice, washed twice with FACS buffer and stained with fluorescently labeled Abs (various concentrations) and Viaprobe (BD Biosciences) or fixable viability dye eFluor 506 or 780 (eBioscience, Hatfield, UK) (to exclude dead cells). FACS analysis of blood samples was performed similarly with an additional blood cell lysis step performed using ammonium chloride solution (Stemcell Technologies, Grenoble, France) according to the manufacturer’s instructions immediately before staining. Abs against the following surface markers were used, with clone names and suppliers given in parentheses: TCRβ (H57-597), CD3 (145-2C11), CD4 (H129.19), CD11b (M1/70), GMCSF (MP1-22E9), IL-17 (TC11-18H10), IFNγ (XMG1.2) (BD Biosciences), CD5 (53-7.3), CD11c (N418), CD207 (Langerin) (EbioL31), MHCII (I-A/I-E) (M5/114.15.2), (eBioscience), CD19 (6D5), EpCAM-1 (2E7) (Biolegend, London, UK) with a variety of conjugated fluorochromes); appropriate isotype controls were purchased from BD Biosciences or eBioscience. For intracellular cytokine staining the BD Biosciences Cytofix/Cytoperm Fixation/Permeabilization Solution Kit with BD GolgiPlug was used according to the manufacturer’s instructions. Briefly, for assessment of cytokine secretion in T cells, 1 × 10^6^ isolated LN cells were cultured for 5 h at 37 °C in complete RPMI in the presence of 500 ng ml^−1^ of ionomycin, 50 ng ml^−1^ of phorbol 12-myristate 13-acetate and Golgistop. To detect GM-CSF production by B cells, LN cells were cultured as described above but in the absence of phorbol 12-myristate 13-acetate and ionomycin. Cultured cells were subsequently stained for surface antigens as above and dead cells were excluded using ethidium monoazide or fixable viability dye eFluor 506 or 780 (eBioscience). Cells were fixed and permeabilized, and stained for intracellular cytokines. The Cytofix/Cytoperm Fixation/Permeabilization Solution Kit was used to stain for CD207. In this case the 5-h culture step was excluded; otherwise the methodology remained the same. Positive populations were defined on the basis of size (to exclude doublet populations), viability (that is, viability dye negative) and ‘fluorescence minus one’ isotype controls. Data were analyzed using FlowJo software (Treestar Inc., Ashland, OR, USA).

### Induction and assessment of EAE

EAE was induced in DBA/1j and C57BL/6 mice with either 50 μg of recombinant rat MOG_1–125_,^41^ emulsified in incomplete Freund’s adjuvant (Sigma-Aldrich) supplemented with 3 mg ml^−1^ of heat-inactivated *Mycobacterium tuberculosis* H37RA (Difco Laboratories, Oxford, UK), or in C57BL/6 mice with 100 μg of MOG_35–55_ peptide (AnaSpec, Fremont, CA, USA) emulsified in incomplete Freund’s adjuvant (Sigma-Aldrich) supplemented with 4 mg ml^−1^ of heat-inactivated *Mycobacterium tuberculosis* H37RA (Difco Laboratories). The emulsion was injected intradermally at the base of the tail. In some experiments (as indicated in the figure legends), animals were treated with 200 ng of pertussis toxin (Enzo Life Sciences, Farmingdale, NY, USA) via intraperitoneal injection on days 0 and 2. Clinical assessment was performed daily by an observer ‘blinded’ to mouse genotype and according to the following criteria: 0, no disease; 1, decreased tail tone; 2, abnormal gait (ataxia) and/or impaired righting reflex (hind limb weakness or partial paralysis); 3, partial hind limb paralysis; 4, complete hind limb paralysis; 5, hind limb paralysis with partial forelimb paralysis; and 6, moribund or dead.

### Generation of mixed chimeric mice

Mice with a mixed WT and ACKR2-deficient hematopoietic compartment were generated using the mixed bone marrow chimera system. WT (CD45.1) mice were irradiated with 5.5 Gy followed by 2 h of rest period and then a second dose of 5.5 Gy. The irradiated mice were reconstituted immediately with a mixed inoculum of bone marrow (50% CD45.1/.2 WT and 50% CD45.2 ACKR2-deficient). The hematopoietic compartment was left for 2 months to repopulate. Chimerism was confirmed by FACS analysis of blood retrieved from the lateral tail vein as described previously using antibodies against mouse CD45.1 (A20) and CD45.2 (104) (eBioscience).

### Immunohistochemistry

Skin was harvested from the injection site immediately proximal to the base of the tail as well as uninvolved skin from the back. The skin samples were embedded in Cryomatrix (Thermo Fisher Scientific, Waltham, MA, USA) and frozen in a bath of isopentane chilled with dry ice. Then, 8 μm frozen tissue sections were cut on a Shandon Crotome FSE (Thermo Fisher Scientific) and stored at −80 °C until required. Tissue sections were fixed in acetone at −20 °C for 20 min. Unless stated otherwise all subsequent steps were performed at room temperature. Tissue sections were rehydrated with PBS for 5 min and blocked with 10% normal goat serum and 3% bovine serum albumin fraction V (Sigma-Aldrich) for 1 h. Sections were stained with unlabeled 5 μg ml^−1^ CD207 (Langerin) (EbioL31) overnight at 4 °C. The sections were then washed twice for 5 min with PBS Tween 0.05% and once for 5 min with PBS alone before staining with 10 μg ml^−1^ goat anti-rat IgG H+L Alexa Fluor 647 (Life Technologies). Wash steps were repeated as previous. Finally, sections were stained with 0.2 μg ml^−1^ DAPI (Life Technologies) solution in PBS for 30 min before mounting using Vectashield (Vector Laboratories, Peterborough, UK). Images were taken on a Zeiss imager M2 microscope and Axiovision 4.8 software (Jena, Germany). The numbers of CD207-positive cells per 10 high- power fields per sample was determined.

### Statistical analysis

GraphPad Prism (La Jolla, CA, USA) was used for all statistical analyses using (as appropriate and as indicated in the figure legends) *t*-test; Mann–Whitney test; Kruskal–Wallis test with Dunn’s multiple comparison post test; one-way analysis of variance with Bonferroni’s multiple comparison post tests; or two-way analysis of variance with repeated measures. To determine correlations, Spearman’s rho test was used. Data are presented as mean±s.d., except for clinical scores that are mean±s.e.m. *P⩽*0.05 was considered statistically significant and all tests were two sided.

## References

[bib1] Rot A, Andrian von UH. Chemokines in innate and adaptive host defense: basic chemokinese grammar for immune cells. Annu Rev Immunol 2004; 22: 891–928. 15032599 10.1146/annurev.immunol.22.012703.104543

[bib2] Nibbs RJB, Graham GJ. Immune regulation by atypical chemokine receptors. Nat Rev Immunol 2013; 13: 815–829. 24319779 10.1038/nri3544

[bib3] Bachelerie F, Ben-Baruch A, Burkhardt AM, Combadiere C, Farber JM, Graham GJ et al. International Union of Basic and Clinical Pharmacology. LXXXIX. Update on the extended family of chemokine receptors and introducing a new nomenclature for atypical chemokine receptors. Pharmacol Rev 2014; 66: 1–79. 24218476 10.1124/pr.113.007724 PMC3880466

[bib4] Nibbs RJ, Kriehuber E, Ponath PD, Parent D, Qin S, Campbell JD et al. The beta-chemokine receptor D6 is expressed by lymphatic endothelium and a subset of vascular tumors. Am J Pathol 2001; 158: 867–877. 11238036 10.1016/s0002-9440(10)64035-7 PMC1850343

[bib5] Madigan J, Freeman DJ, Menzies F, Forrow S, Nelson SM, Young A et al. Chemokine scavenger D6 is expressed by trophoblasts and aids the survival of mouse embryos transferred into allogeneic recipients. J Immunol 2010; 184: 3202–3212. 20147628 10.4049/jimmunol.0902118

[bib6] Martinez de la Torre Y, Buracchi C, Borroni EM, Dupor J, Bonecchi R, Nebuloni M et al. Protection against inflammation- and autoantibody-caused fetal loss by the chemokine decoy receptor D6. Proc Natl Acad Sci USA 2007; 104: 2319–2324. 17283337 10.1073/pnas.0607514104 PMC1892950

[bib7] Nibbs RJ, Wylie SM, Yang J, Landau NR, Graham GJ. Cloning and characterization of a novel promiscuous human beta-chemokine receptor D6. J Biol Chem 1997; 272: 32078–32083. 9405404 10.1074/jbc.272.51.32078

[bib8] McKimmie CS, Fraser AR, Hansell C, Gutiérrez L, Philipsen S, Connell L et al. Hemopoietic cell expression of the chemokine decoy receptor D6 is dynamic and regulated by GATA1. J Immunol. 2008; 181: 3353–3363. 18714007 10.4049/jimmunol.181.5.3353

[bib9] Hansell CAH, Schiering C, Kinstrie R, Ford L, Bordon Y, McInnes IB et al. Universal expression and dual function of the atypical chemokine receptor D6 on innate-like B cells in mice. Blood 2011; 117: 5413–5424. 21450903 10.1182/blood-2010-11-317115 PMC3188399

[bib10] Rauch PJ, Chudnovskiy A, Robbins CS, Weber GF, Etzrodt M, Hilgendorf I et al. Innate response activator B cells protect against microbial sepsis. Science 2012; 335: 597–601. 22245738 10.1126/science.1215173 PMC3279743

[bib11] Bonecchi R, Locati M, Galliera E. Differential recognition and scavenging of native and truncated macrophage-derived chemokine (macrophage-derived chemokine/CC chemokine ligand 22) by the D6 decoy receptor. J Immunol 2004; 172: 4972–4976. 15067078 10.4049/jimmunol.172.8.4972

[bib12] Weber M, Blair E, Simpson CV, O'Hara M, Blackburn PE, Rot A et al. The chemokine receptor D6 constitutively traffics to and from the cell surface to internalize and degrade chemokines. Mol Biol Cell 2004; 15: 2492–2508. 15004236 10.1091/mbc.E03-09-0634 PMC404040

[bib13] Fra AM, Locati M, Otero K, Sironi M, Signorelli P, Massardi ML et al. Cutting edge: scavenging of inflammatory CC chemokines by the promiscuous putatively silent chemokine receptor D6. J Immunol 2003; 170: 2279–2282. 12594248 10.4049/jimmunol.170.5.2279

[bib14] Galliera E, Jala VR, Trent JO, Bonecchi R, Signorelli P, Lefkowitz RJ et al. beta-Arrestin-dependent constitutive internalization of the human chemokine decoy receptor D6. J Biol Chem 2004; 279: 25590–25597. 15084596 10.1074/jbc.M400363200

[bib15] McCulloch CV, Morrow V, Milasta S, Comerford I, Milligan G, Graham GJ et al. Multiple roles for the C-terminal tail of the chemokine scavenger D6. J Biol Chem 2008; 283: 7972–7982. 18201974 10.1074/jbc.M710128200

[bib16] Di Liberto D, Locati M, Caccamo N, Vecchi A, Meraviglia S, Salerno A et al. Role of the chemokine decoy receptor D6 in balancing inflammation, immune activation, and antimicrobial resistance in Mycobacterium tuberculosis infection. J Exp Med 2008; 205: 2075–2084. 18695004 10.1084/jem.20070608 PMC2526202

[bib17] Jamieson T, Cook DN, Nibbs RJB, Rot A, Nixon C, McLean P et al. The chemokine receptor D6 limits the inflammatory response in vivo. Nat Immunol 2005; 6: 403–411. 15750596 10.1038/ni1182

[bib18] Martinez de la Torre Y, Locati M, Buracchi C, Dupor J, Cook DN, Bonecchi R et al. Increased inflammation in mice deficient for the chemokine decoy receptor D6. Eur J Immunol 2005; 35: 1342–1346. 15789340 10.1002/eji.200526114

[bib19] Berres M-L, Trautwein C, Zaldivar MM, Schmitz P, Pauels K, Lira SA et al. The chemokine scavenging receptor D6 limits acute toxic liver injury in vivo. Biol Chem 2009; 390: 1039–1045. 19642876 10.1515/BC.2009.119

[bib20] Nibbs RJB, Gilchrist DS, King V, Ferra A, Forrow S, Hunter KD et al. The atypical chemokine receptor D6 suppresses the development of chemically induced skin tumors. J Clin Invest 2007; 117: 1884–1892. 17607362 10.1172/JCI30068 PMC1904306

[bib21] Whitehead GS, Wang T, DeGraff LM, Card JW, Lira SA, Graham GJ et al. The chemokine receptor D6 has opposing effects on allergic inflammation and airway reactivity. Am J Respir Crit Care Med 2007; 175: 243–249. 17095748 10.1164/rccm.200606-839OC PMC1899265

[bib22] Ansel KM, Harris RBS, Cyster JG. CXCL13 is required for B1 cell homing, natural antibody production, and body cavity immunity. Immunity 2002; 16: 67–76. 11825566 10.1016/s1074-7613(01)00257-6

[bib23] Szekanecz Z, Vegvari A, Szabo Z, Koch AE. Chemokines and chemokine receptors in arthritis. Front Biosci 2010; 2: 153–167. 10.2741/s53 PMC2917905 20036936

[bib24] Proudfoot AEI, de Souza ALS, Muzio V. The use of chemokine antagonists in EAE models. J Neuroimmunol 2008; 198: 27–30. 18550179 10.1016/j.jneuroim.2008.04.007

[bib25] Schall TJ, Proudfoot AEI. Overcoming hurdles in developing successful drugs targeting chemokine receptors. Nat Rev Immunol 2011; 11: 355–363. 21494268 10.1038/nri2972

[bib26] Liu L, Graham GJ, Damodaran A, Hu T, Lira SA, Sasse M et al. Cutting edge: the silent chemokine receptor D6 is required for generating T cell responses that mediate experimental autoimmune encephalomyelitis. J Immunol 2006; 177: 17–21. 16785491 10.4049/jimmunol.177.1.17

[bib27] Lee KM, McKimmie CS, Gilchrist DS, Pallas KJ, Nibbs RJ, Garside P et al. D6 facilitates cellular migration and fluid flow to lymph nodes by suppressing lymphatic congestion. Blood 2011; 118: 6220–6229. 21979941 10.1182/blood-2011-03-344044 PMC3234674

[bib28] Sonderegger I, Iezzi G, Maier R, Schmitz N, Kurrer M, Kopf M. GM-CSF mediates autoimmunity by enhancing IL-6-dependent Th17 cell development and survival. J Exp Med 2008; 205: 2281–2294. 18779348 10.1084/jem.20071119 PMC2556786

[bib29] Nandakumar K, Holmdahl R. Antibody-induced arthritis: disease mechanisms and genes involved at the effector phase of arthritis. Arthritis Res Ther 2006; 8: 223. 17254316 10.1186/ar2089 PMC1794524

[bib30] Vetrano S, Borroni EM, Sarukhan A, Savino B, Bonecchi R, Correale C et al. The lymphatic system controls intestinal inflammation and inflammation-associated Colon Cancer through the chemokine decoy receptor D6. Gut 2010; 59: 197–206. 19846409 10.1136/gut.2009.183772

[bib31] Singh MD, King V, Baldwin H, Burden D, Thorrat A, Holmes S et al. Elevated expression of the chemokine-scavenging receptor D6 is associated with impaired lesion development in psoriasis. Am J Pathol 2012; 181: 1158–1164. 22867710 10.1016/j.ajpath.2012.06.042 PMC3532592

[bib32] Constant S, Sant'Angelo D, Pasqualini T, Taylor T, Levin D, Flavell R et al. Peptide and protein antigens require distinct antigen-presenting cell subsets for the priming of CD4+ T cells. J Immunol 1995; 154: 4915–4923. 7730604

[bib33] Constant S, Schweitzer N, West J, Ranney P, Bottomly K. B lymphocytes can be competent antigen-presenting cells for priming CD4+ T cells to protein antigens in vivo. J Immunol 1995; 155: 3734–3741. 7561077

[bib34] O'Neill SK, Shlomchik MJ, Glant TT, Cao Y, Doodes PD, Finnegan A. Antigen-specific B cells are required as APCs and autoantibody-producing cells for induction of severe autoimmune arthritis. J Immunol 2005; 174: 3781–3788. 15749919 10.4049/jimmunol.174.6.3781

[bib35] Weber MS, Prod'homme T, Patarroyo JC, Molnarfi N, Karnezis T, Lehmann-Horn K et al. B-cell activation influences T-cell polarization and outcome of anti-CD20 B-cell depletion in central nervous system autoimmunity. Ann Neurol 2010; 68: 369–383. 20641064 10.1002/ana.22081 PMC3375897

[bib36] Matsushita T, Yanaba K, Bouaziz J-D, Fujimoto M, Tedder TF. Regulatory B cells inhibit EAE initiation in mice while other B cells promote disease progression. J Clin Invest 2008; 118: 3420–3430. 18802481 10.1172/JCI36030 PMC2542851

[bib37] Attanavanich K, Kearney JF. Marginal zone, but not follicular B cells, are potent activators of naive CD4 T cells. J Immunol 2004; 172: 803–811. 14707050 10.4049/jimmunol.172.2.803

[bib38] Zhong X, Gao W, Degauque N, Bai C, Lu Y, Kenny J et al. Reciprocal generation of Th1/Th17 and T(reg) cells by B1 and B2 B cells. Eur J Immunol 2007; 37: 2400–2404. 17683116 10.1002/eji.200737296

[bib39] MacLellan LM, Montgomery J, Sugiyama F, Kitson SM, Thummler K, Silverman GJ et al. Co-opting endogenous immunoglobulin for the regulation of inflammation and osteoclastogenesis. Arthritis Rheum 2011; 63: 3897–3907. 22127707 10.1002/art.30629 PMC3598489

[bib40] Joosten LA, Lubberts E, Durez P, Helsen MM, Jacobs MJ, Goldman M et al. Role of interleukin-4 and interleukin-10 in murine collagen-induced arthritis. Protective effect of interleukin-4 and interleukin-10 treatment on cartilage destruction. Arthritis Rheum 1997; 40: 249–260. 9041936 10.1002/art.1780400209

[bib41] Adelmann M, Wood J, Benzel I, Fiori P, Lassmann H, Matthieu JM et al. The N-terminal domain of the myelin oligodendrocyte glycoprotein (MOG) induces acute demyelinating experimental autoimmune encephalomyelitis in the Lewis rat. J Neuroimmunol 1995; 63: 17–27. 8557821 10.1016/0165-5728(95)00124-7

