# Supplementary figures and images for: The atypical chemokine receptor ACKR2 suppresses Th17 responses to protein autoantigens
Source: Immunol Cell Biol. 2014 Oct 28;93(2):167–76. doi: 10.1038/icb.2014.90 (PMC4340511; doi:10.1038/icb.2014.90)

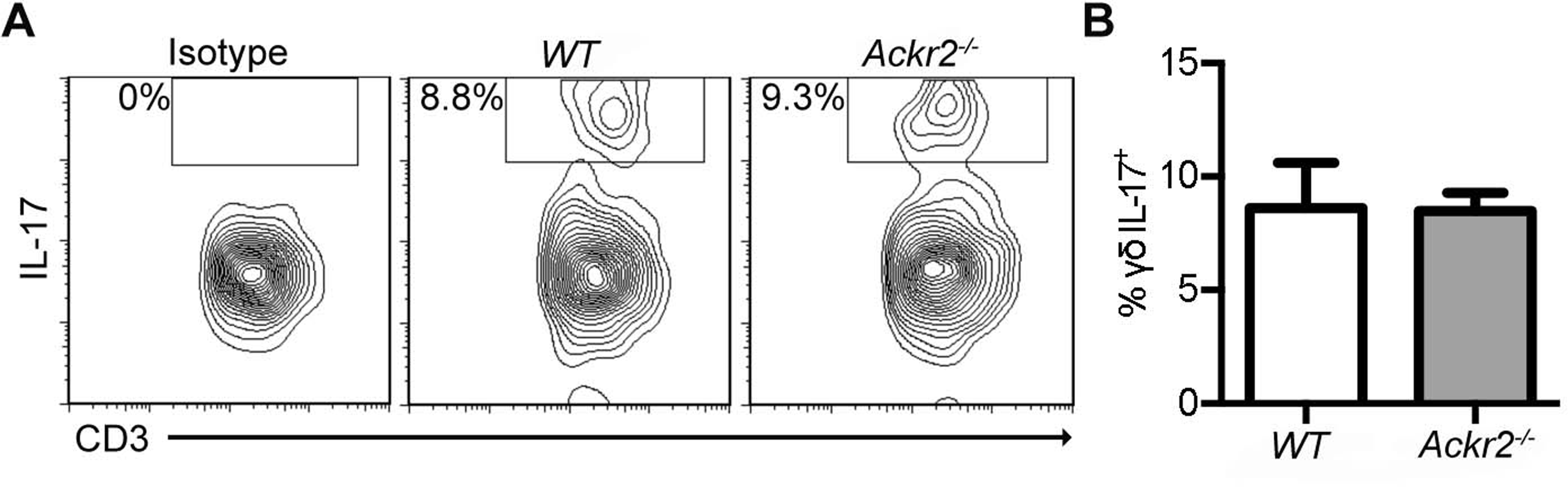

Supplement: Supplementary Figure 1 [file icb201490x1.tif]

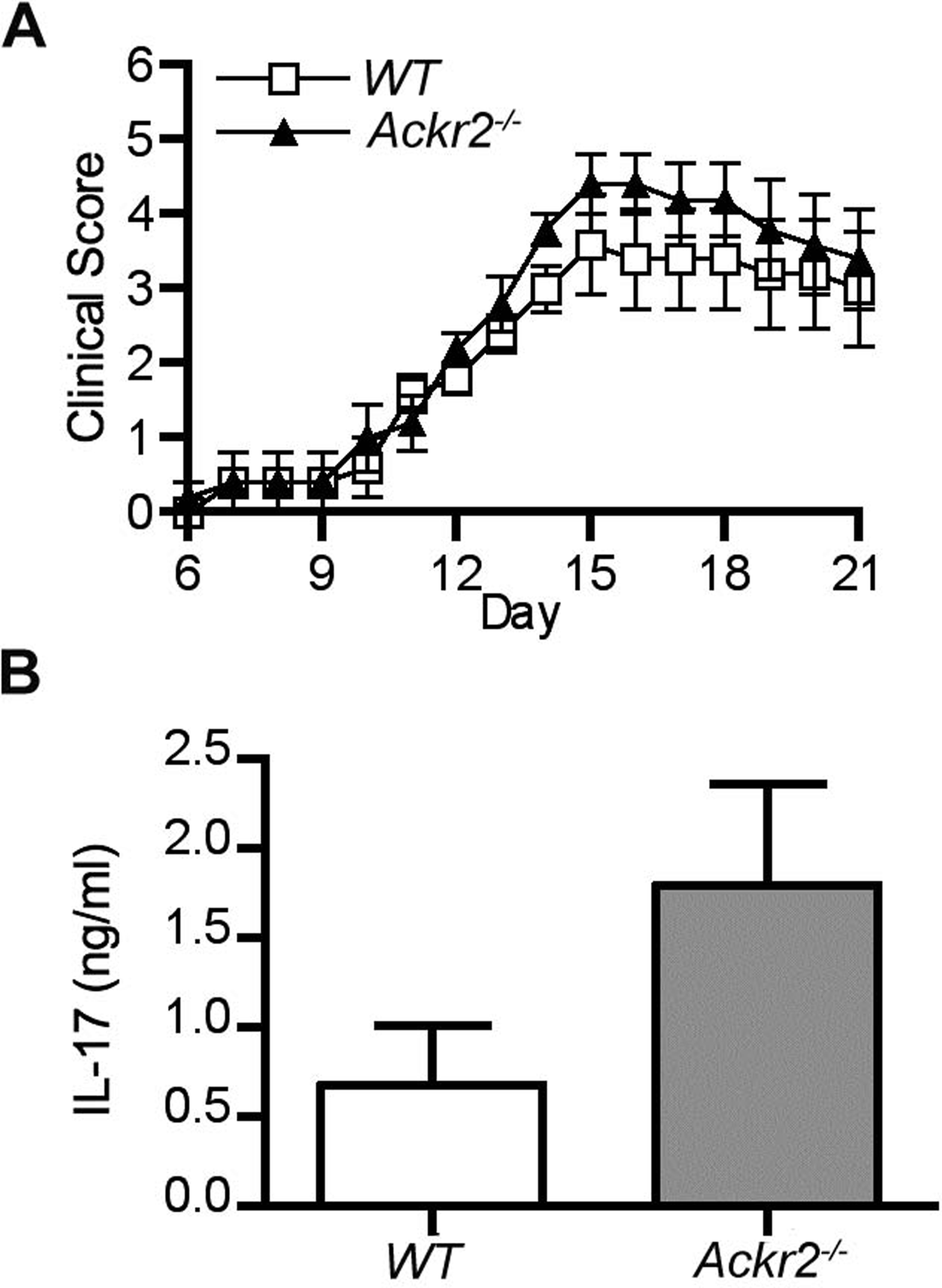

Supplement: Supplementary Figure 2 [file icb201490x2.tif]
